# Comparison of mortality in patients on chemotherapy or immunotherapy during and before COVID-19 pandemic. Multicenter matched cohort study in Argentina

**DOI:** 10.17843/rpmesp.2023.402.12519

**Published:** 2023-06-30

**Authors:** Boris Itkin, Samanta Straminsky, Hernán Cáceres, Mariana Onassis, Agustín Emilio García, Laura Avanzi, Lucia Kaminszczik, Richard Serna Sejas, Mara Rapaccioli, Gustavo Billordo, Damián Lavaccara, Julián Lanzavecchia, Luz Gibbons, Eugenia Settecase, Ariel Bardach

**Affiliations:** 1 Hospital Juan A. Fernández, Buenos Aires, Argentina. Hospital Juan A. Fernández Buenos Aires Argentina; 2 Sanatorio Luis Pasteur, Catamarca, Argentina. Sanatorio Luis Pasteur Catamarca Argentina; 3 Sanatorio Dr. Julio Méndez, Buenos Aires, Argentina. Sanatorio Dr. Julio Méndez Buenos Aires Argentina; 4 Policlínico Modelo de Cipolletti, Rio Negro, Argentina. Policlínico Modelo de Cipolletti Rio Negro Argentina; 5 Center for Research in Epidemiology and Public Health (CIESP) Instituto de Efectividad Clínica y Sanitaria (IECS-CONICET), Buenos Aires, Argentina. Center for Research in Epidemiology and Public Health (CIESP) Instituto de Efectividad Clínica y Sanitaria (IECS-CONICET) Buenos Aires Argentina

**Keywords:** Cancer, Chemotherapy, Immunotherapy, COVID-19, Mortality, Cohort Studies

## Abstract

**Objectives.:**

To compare all-cause mortality of unvaccinated oncology patients who received chemotherapy or immunotherapy during the pandemic with those treated before the pandemic.

**Materials and methods.:**

We conducted a cohort study in four tertiary hospitals in Argentina. Outpatients with a solid neoplasm of any stage under-going cytotoxic or intravenous immunotherapy were eligible. The pandemic cohort was enrolled during the initial phase of the outbreak and compared with a pre-pandemic cohort using propensity score matching (PSM). Subjects were matched for age, sex, health insurance, risk factors for severe COVID-19 complications, performance status, cancer type and treatment, line of treatment, and body mass index. All-cause mortality was estimated for both cohorts after 6 months of follow-up.

**Results.:**

A total of 169 patients were recruited between April and August 2020 for the pandemic cohort and 377 for the pre-pandemic cohort in the same months of 2019; 168 patients were matched. After PSM, all-cause mortality was 17.9% in the pandemic cohort and 18.5% in the pre-pandemic cohort; the Relative Risk was 0.97 (95 % confidence interval: 0.61-1.52; p=0.888). In the pandemic cohort, 30/168 patients died, but none from COVID-19.

**Conclusions.:**

Our findings show that the mortality rate of unvaccinated ambulatory patients on active intravenous oncology treatment during the COVID-19 pandemic did not increase.

## INTRODUCTION

Cancer is the second leading cause of mortality in Latin America and The Caribbean (LAC), with over 700,000 deaths in 2020 ^1^. Significant difficulties already posed to the region by this burden were further exacerbated by the COVID-19 pandemic contingency ^2^. The entire spectrum of cancer care, from prevention to survivorship, has been affected by the COVID-19 pandemic, presenting a new challenge to providers regarding weighing the benefits of anti-cancer treatments and the risk of their administration amidst the pandemic. An early study showed that intravenous (IV) chemotherapy was associated with an increased risk of severe complications in cancer patients infected with SARS-CoV-2 ^3^. Chemotherapy and disease-induced immunosuppression, treatment-related pulmonary toxicity, the need for hospitalization with the associated risk of nosocomial infections alongside the saturation of the health system, social distancing, and economic issues have been suggested as factors potentially influencing the risk of severe complications and death of cancer patients on active anticancer treatment ^4^^-^^6^. This was reflected in the recommendations from medical societies and experts suggesting the substitution of chemotherapy with endocrine drugs, the use of treatment protocols with a lower number of cycles, and replacing of IV drugs with oral analogs leading to modifications of the management practice with potential impact on oncological outcomes ^4^^,^^5^^,^^7^^,^^8^. During the pandemic, there was also a concern regarding the safety of immunotherapy due to a significant incidence of immune-related pneumonitis requiring corticosteroids and immunosuppressors ^6^^,^^9^.

Many studies in the field of COVID-19 and cancer have focused on outcomes of oncological patients with proven COVID-19 infection compared to those without cancer or COVID-19 infection. These studies usually used data from COVID-19 positive subjects ^10^^,^^11^. However, this approach may be less appropriate for assessing the incremental risk of death associated with the COVID-19 pandemic in the general population of ambulatory cancer patients receiving an active IV treatment. On the other hand, the estimation of COVID-19 specific mortality as an indicator of the pandemic impact on oncological patients’ health may be challenging or unreliable due, for instance, to cause of death misclassification, variations in COVID-19 tests procedures and reporting, and the disease displacement phenomenon ^12^. All-cause mortality is a measure that encompasses all death from any cause and allows to overcome the cited issues providing a metric of pandemic impact on overall mortality ^12^.

Most research on COVID-19 and cancer is carried out in developed countries ^12^. However, it is not clear to what extent the results of these studies can be applied to developing countries. In LAC, the pre-pandemic healthcare landscape was already rife with fragmented and underfunded healthcare systems, inequities in access to quality care, and reduced availability of COVID-19 tests and vaccines. Across the region, lockdowns and quarantines were widely implemented ^13^. We aimed to investigate whether all-cause mortality of ambulatory oncological patients receiving active IV cytotoxic drugs or Immune checkpoint inhibitors (ICI) increased during the pandemic compared to the pre-pandemic period.

**KEY MESSAGES**

Motivation for the study. The impact of the COVID-19 pandemic on the risk of death in cancer patients on chemotherapy and immunotherapy is controversial. Published studies mainly compared patients on anti-cancer therapy to those off treatment or COVID-19 positive cancer patients to COVID-19 negative ones. Few studies were conducted in developing countries.

Main findings. Mortality didn’t increase in unvaccinated outpatients on active intravenous oncology treatment during the COVID-19 pandemic.

Implications. This is the first propensity score-matched cohort study evaluating the impact of the COVID-19 pandemic on the population of unvaccinated oncology patients receiving intravenous anticancer therapy.

## MATERIALS AND METHODS

### Design and Setting

We conducted a multicenter prospective cohort study that used a propensity score-matched retrospective cohort from the year before the pandemic as reference. We formed a collaborative group involving four tertiary hospitals in Argentina, one public and three private, which were located in different geographic regions. We prospectively recruited patients for the pandemic cohort in the Day Care Units of the participating centers between April 15, 2020, and August 26, 2020. The reference cohort (pre-pandemic cohort) was built using the data from medical records of patients treated in the same centers in the matched period of 2019 before the onset of the pandemic.

### Participants

To ensemble the pandemic cohort, we used a simple probabilistic sampling within each center. Eligible subjects were randomly assigned to participate or not using a random numbers generator in a virtual remote randomization office. We used convenience sampling for the historical cohort. Patients of any age and sex with a histologically proven diagnosis of a solid neoplasm who received IV cytotoxic or ICI therapy as a single agent or in any combination were eligible regardless of the availability of the COVID-19 assay and its results. Patients on active concurrent chemoradiation treatment and those receiving a combination of IV chemotherapy and oral drugs, either target or cytotoxic, were also included. We excluded patients who received hormone or target therapy without concomitant cytotoxic drugs or ICIs. The follow-up period was six months.

### Sample size

At the time of the study design, in March 2020, reports showed a significant increase in serious clinical events in COVID-19 positive cancer patients who received chemotherapy compared to those who did not, with an Odds Ratio of 5.34 (95% confidence interval [95%CI ] 1.8 -16.2) ^3^. As no other relevant study on the topic was available then, we took this CI’s lower limit as reference. With 168 patients per cohort, the study had a statistical power of 87% to detect a 70% increase in mortality in the pandemic cohort compared with the pre-pandemic period.

### Data source and measurements

We collected data on clinical, demographic, treatment-related characteristics, and survival outcomes from (physical and electronic) medical records. Patients and their relatives were contacted by phone when needed. Tumors were staged according to the American Joint Committee on Cancer Staging Manual, 8th Edition ^14^. Due to a significant shortage of COVID-19 swabs and serological tests in Argentina at the time of study design in March 2020 and until the end of the follow-up period, these tests were not mandatory but performed according to medical prescriptions and availability. No patient received the COVID-19 vaccine as the follow-up period ended before it became available in Argentina.

### Variables

*Exposure and outcomes*

The COVID-19 status of any individual at any point in time cannot be known, and survival is not just influenced by the clinical severity of the COVID-19 disease but rather potentially depends on a complex interplay of many factors that are highly variable in time and therefore, difficult to estimate. They include the possibility of COVID-19 contagion, recovery from complications of anticancer treatment and COVID-19 infection, oncological treatment delays or discontinuation, availability of health care system resources, family and social support, individual adherence to restriction measures, and the capacity to afford high health-related costs among others. Therefore, we defined patients’ “immersion” into the pandemic environment as exposure.

The primary outcome of our study was mortality from any cause at the end of the follow-up period in both cohorts, defined as the number of deaths divided by the total number of participants. The rate of severe complications was the secondary outcome. Time-dependent survival was not calculated because individual data points were unavailable in the pre-pandemic cohort.

*Cohorts matching and covariates*

We used propensity score matching (PSM) to control for known confounders because of possible differences between cohorts regarding covariates of potential prognostic significance. For model specification, we previously performed a Cox regression analysis using the survival data from the pandemic cohort after four months of the follow-up. We found variables that were significantly associated with survival probability: Eastern Cooperative Oncology Group (ECOG) Performance Status, treatment line, and body mass index (data available on request). In addition, we included covariates that can potentially influence the likelihood of death, such as age, sex, type of health insurance, number of medications, and tumor type. In order to assess prognosis differences according to cancer subtype, we classified tumors into three prognostic groups according to expected median 5-year survival at all stages (Table 1) ^15^^,^^16^. Regarding the tumor stage, we assumed that the short-term probability of death could be accounted for by dividing patients into non-metastatic and advanced disease categories. In this case, the variable “Treatment line” includes the relevant prognostic information related to the tumor stage since neoadjuvant treatments match non-metastatic stages. The list of covariates included in the propensity score is shown in Supplementary Material.

**Table 1:** Prognostic groups by cancer subtype

Prognostic group	Cancer subtype
1	Testicular, Prostate, Breast, GIST
2	Anal, Penile, Cervical, Colorectal, Soft tissue and bone sarcomas
3	Biliopancreatic, Hepatocarcinoma, Lung, Mesothelioma, Esophageal
4	Melanoma, Endometrial Renal, Head and Neck, Bladder
5	Ovarian, vulvovaginal, non-melanoma skin
6	Gastric, CNS

GIST: Gastrointestinal stromal tumor, CNS: Central Nervous System

### Statistical analysis

There was a moderate percentage (7.5%) of missing BMI values, whereas the remaining covariates showed a low percentage of missing data (0.0%-0.5%). We imputed missing data with the multiple imputations by chained equations (MICE) method because of its universal acceptance, flexibility, and good performance with continuous and categorical variables ^17^. We started by identifying the variables that had missing values and creating chains of equations for each of these variables. These chains consist of regression models that predict a missing value in a variable based on the observed values in other variables. We iteratively updated the imputations for each variable by sequential sampling from the conditional distributions of the other variables in the chain. This process was repeated multiple times to ensure the convergence and stability of the imputations.

Propensity scores were estimated using logistic regression ^18^. We used optimal one-to-one matching with a replacement method based on the Ford-Fulkerson network flow optimization algorithm as it is expected to outperform the one-to-one greedy matching ^19^^-^^21^. We used standardized mean difference (SMD) and density plots to assess the group balance. Since the SMD between the matched groups was below the threshold of 0.1, we concluded that the balance was satisfactory. The matched cohorts were compared with a chi-squared test. We used the Matchlt package for the application of the method ^22^.

We reported the mortality rate of both cohorts and the crude and adjusted relative risk (RR) with a 95% confidence interval. We estimated the adjusted RR using logistic regression with Poisson distribution log link function. We adjusted for the variables included in the PSM. We used the binomial method to estimate severe complications rate with 95%CI. All statistical tests were done at a 0.05 alpha using the open-source R software 4.0.3.

### Ethical aspects

The Ethics Committees of all participating centers approved the study (date: 08/04/ 2020, registration code: 1234, number: DI-2020-353-CABA-HGAJF), and all patients in the pandemic cohort signed an informed consent form.

## RESULTS

*Cohorts and matching*

**Figure1. Patients:**
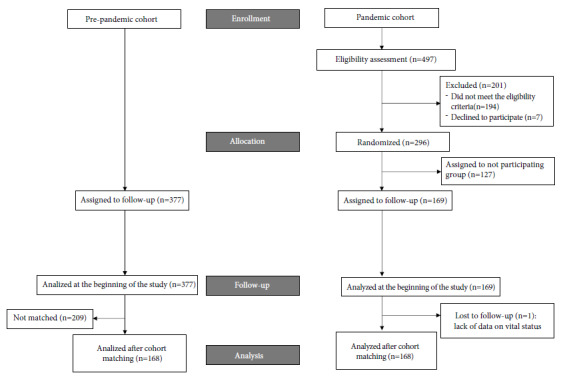
flow in the study.

Before PSM, the number of patients in the pandemic and pre-pandemic cohorts were 169 and 377, respectively (Figure 1). At baseline, the mean age was 59.2 years (standard deviation [SD ]: 13.7 years) in the pandemic cohort and 59.4 years (SD: 13.5 years) in the pre-pandemic cohort. The ratio of women was 62.7% and 68.7% in both cohorts, respectively. Chemotherapy was the most common treatment (92.6% in the pandemic cohort and 95.5% in pre-pandemic cohort), and a small proportion of patients had received ICI (7.2% and 4.5%, respectively). Neoadjuvant and first-line therapies were the most frequent with 78.7%, and 77.3%, respectively. The proportion of patients with two or more risk factors for severe complications of COVID-19 infection was 32.1% and 32.7%, respectively. The clinical, demographic, and treatment-related characteristics of the participants are summarized in Supplementary Material. The median follow-up time was 6.0 months in both cohorts. In the pandemic cohort, 4.8% had a nasal swab positive for COVID-19. One out of 169 patients from the pandemic cohort was lost from follow-up and did not have data on the main outcome. None out of 377 patients was lost from follow-up in the pre-pandemic cohort.

As a result of PSM, 168 subjects were matched. In the pre-pandemic cohort, 209 subjects were left out while only one subject was dropped from the pandemic cohort. Patients’ characteristics in pandemic and pre-pandemics cohorts after matching are shown in Table 2. No statistically significant difference was found between cohorts by covariates included in the model. The distribution of propensity scores in both matched cohorts and unmatched subjects is shown in Supplementary Material. The absolute SMD for each covariate is shown in Supplementary Material.

**Table 2:** Comparison of cohorts after matching.

Variables	Cohort				p-value
	Pandemic (N=168)		Pre-pandemic (N=168)		
	n	%	n	%	
Age					
65 or younger	101	60.1	100	59.5	1.000
Older than 65	67	39.9	68	40.5	
Sex					
Male	62	36.9	66	39.3	0.736
Female	106	63.1	102	60.7	
Health insurance type					
General public health	25	14.9	22	13.1	0. 811
Healthcare for the elderly	34	20.2	38	22.6	
Private healthcare	109	64.9	108	64.3	
Treatment type					
Chemotherapy	156	92.9	154	91.7	0.838
Immune therapy	12	7.1	14	8.3	
Tumor type group					
1 ^a^	55	32.7	48	28.6	0.709
2,4,5 ^b^	77	45.8	82	48.8	
3,6 ^c^	36	21.4	38	22.6	
Treatment line					
Neoadjuvant	76	45.2	74	44.0	0.924
First line	57	33.9	56	33.3	
Second or third	35	20.8	38	22.6	
Number of drugs					
One or two	159	94.6	157	93.5	0.818
Three or more	9	5.4	11	6.5	
COVID-19 risk factors					
None	54	32.1	47	28.0	0.677
1^d^	60	35.7	66	39.3	
2-6 ^d^	54	32.1	55	32.7	
ECOG					
0	50	29.8	50	29.8	0.939
1	100	59.5	98	58.3	
2-4	18	10.7	20	11.9	
Obesity					
No	132	78.6	134	79.8	0.893
Yes	36	21.4	34	20.2	

ECOG: Eastern Cooperative Oncology Group.

a Testicular, prostate, breast, gastrointestinal stromal tumors.

b Anal, penile, cervical, colorectal, soft tissue and bone sarcomas, melanoma, renal, head and neck, bladder, ovarian, vulvovaginal, non-melanoma skin cancer.

c Biliopancreatic cancer, hepatocarcinoma, lung, mesothelioma, esophageal, gastric, central nervous system.

d Number of COVID-19 risk factors: smoking or former smoking, respiratory disease, hypertension, heart disease, diabetes, immunodeficiency/chronic corticosteroid use, chronic kidney disease, chronic liver disease.

*Mortality and admissions*

After PSM, the all-cause mortality rate was 30/168 (17.9%, 95%CI: 12.4% - 24.5%) in the pandemic cohort and 31/168 (18.5%, 95%CI: 12.9% - 25.2%) in the pre-pandemic cohort (RR: 0.97, 95%CI: 0.61 - 1.52; p=0.888 and adjusted RR [aRR ]: 1.07, 95%CI: 0.63 - 1.79; p=0.810). The rate of serious complications in the pandemic cohort was 8/167 (4.8%, 95%CI: 2.1% - 9.2%). Of the 30 patients who died in the pandemic cohort, 29 died from cancer progression, 0 patients (0.0%, 95%CI: 0.0% - 1.2%) from SARS-CoV-2 virus infection, and one patient (3.3%, 95%CI: 0.0% - 1.7%) from another cause. No statistically significant association was found between vital status at the end of follow-up and COVID-19 positivity (Fisher’s exact test p-value equal to 1). In the pandemic cohort, 8/167 (4.8%, 95%CI: 2.1% - 9.2%) patients were admitted to an Intensive Care Unit (ICU), 7/167 (4.2%, 95%CI: 1.7% - 8.4%) to a High Dependency Unit, and 47/167 (28.1%, 95%CI: 21.5% - 35.6%) to an ordinary ward. No statistically significant relationship was found between admissions and COVID-19 test positivity (Fisher’s exact test p-value equal to 1).

*Sensitivity Analysis*

To assess whether our results were influenced by the inclusion of patients on immunological treatment, we performed a sensitivity analysis by eliminating them from the cohorts. After PSM, all-cause mortality was 17.9% (28/56) and 17.5% (27/154) in the pandemic and pre-pandemic cohorts, respectively; aRR: 1.07 (95% CI: 0.57- 1.99; p=0.329).

*Missing data*

BMI data had a moderate proportion (7.5%) of missing values. On the other hand, the rest of covariates had significantly fewer missing values (ranging from 0.0% to 0.5%).

## DISCUSSION

Unlike most published studies that usually focus on the outcomes of patients with COVID-19 and cancer, we addressed the risk of IV cancer therapy in the pandemic context differently. We compared the mortality rate of oncological patients on active IV chemotherapy or immunotherapy regardless of their COVID-19 status to a matched cohort from the pandemic and pre-pandemic period. The study was conducted in a developing country before vaccine use ^23^.

After adjusting for potential confounders (age, sex, health insurance, drug type, primary tumor group, line of treatment, number of drugs, risk factors, ECOG functional status and obesity), we found no statistically significant difference in the all-cause mortality rate of patients treated during the pandemic (17.9%) versus those treated before the pandemic (18.5%) (RR: 0.97; 95%CI: 0.61-1.52, p=0.888).

We found a mortality rate of 18% at 6-months of follow-up, which is higher than the 4% to 8% rate reported by a large cohort study from the United Kingdom (UK). This British study included patients on target drugs and oral chemotherapy who progressed favorably, which could be a possible explanation. However, differences in the quality of care cannot be ruled out ^24^.

To date, many studies with differences in their design, size, population, follow-up time, definitions, diagnostic methods, and events of interest assessed mortality in cancer patients during the pandemic. Many were retrospective, comparing patients with COVID-19 and cancer, who were on anti-cancer therapy to those without any active treatment. Several systematic reviews attempted to summarize the available evidence. Meta-analyses by Wang *et al.*, Yekeduz *et al.*, Park *et al.*, and Wu *et al.*, included mostly retrospective studies from China, Europe, and Northern America with partial overlap ^25^^-^^27^. These studies compared mortality in patients with COVID-19 with solid and hematological neoplasms on chemotherapy with the control arm, typically by combining patients who were receiving cancer treatment with those receiving non-cytotoxic treatment. All of them found increments in mortality in the chemotherapy arm. In contrast, Lin *et al.*, concluded that chemotherapy and ICI did not increase mortality in patients with cancer and COVID-19 ^28^^,^^29^. 

A large study from the UK by Russel *et al*., which is similar to our study, used a cohort from a pre-pandemic period (2019) as a control group ^30^. Besides patients on cytotoxic drugs and immunotherapy, the study included patients who received biological and targeted therapy. The cohorts were not balanced by PSM. The authors found that systemic chemotherapy during the first wave of the COVID-19 pandemic did not increase the mortality in patients with solid tumors compared to those during the pre-pandemic period. Low COVID-19 infection and mortality rates were reported by the research, which is similar to our findings ^30^.

To reconcile the controversial conclusions of previously published analyses, we hypothesize that even though cytotoxic drugs still may increase mortality in the general population of cancer patients, it could be difficult to detect this effect in environments with low prevalence and transmission rates. The type of neoplasm, hematological or solid, can also be a factor. A large study would be necessary to test this hypothesis. Researchers should consider designing future studies with greater data granularity to better assess relevant subgroups.

In our study, the absence of excess mortality in the pandemic cohort, which is different with many previous publications, could provide potential explanations. For example, the overall impact of the COVID-19 pandemic in the general population of cancer patients may be less than in those with clinically overt infection or positive COVID-19 tests. Most published studies evaluated the latter populations. If SARS-CoV-2 virus infection in the general population of patients with solid tumors is predominantly asymptomatic, overall mortality will not increase unless the infection is highly lethal. In this sense, the low pandemic burden in Argentina during the study period might be a possible explanation. However, more than 2 million cases of SARS-CoV-2 virus infection were reported in Argentina during the mentioned period ^31^. The true cumulative number of cases may be even higher, considering that the capacity for analysis was limited even in high-income countries. ^32^.

The strengths of our study are the prospective enrollment in the pandemic cohort, the inclusion of multiple centers widely distributed across the national territory, the inclusion of both private and public healthcare systems, and the cohort matching by propensity score. Our study has several limitations. The comparison of a prospectively enrolled cohort with a retrospective one is the main source of bias and the main limitation of our study. The utilization of PSM is a way in which we sought to reduce bias. Another important limitation is the small sample size. The study was designed to detect only large increments in mortality as the assumptions made during the design process at the very beginning of the pandemic were influenced by the information of a potentially very significant impact of COVID-19 on oncological patients’ health ^3^^,^^5^. The proportion of subjects tested for COVID-19 was low. Which is probably due to the significantly reduced test availability in Argentina during the first phase of the pandemic alongside a low rate of symptomatic COVID-19 infection in our study. We hypothesize that the latter could be a result of reduced viral spread in the study population because of massive and strict lockdowns, although their adherence gradually diminished over time due to fatigue ^13^. The short follow-up period is another limitation of our work. The follow-up was discontinued once vaccination started in Argentina. Our findings may not be representative of the entire population of cancer patients or applicable to different geographic regions or time periods.

According to a review conducted by the authors of this paper, this is the first propensity score-matched cohort study evaluating the impact of the COVID-19 pandemic on unvaccinated oncology patients receiving IV anticancer therapy compared to those during a non-pandemic period. Despite the discussed limitations, our study provides some empirical support for the idea that IV chemotherapy and immunotherapy could be safely administered during the pandemic prior to the use of COVID-19 vaccine in a middle-income country and contributes to the debate on the interaction between the COVID-19 pandemic and oncology care. Our results suggest that, even in the midst of a pandemic, it remains crucial to prioritize and ensure adequate access to non-COVID-19 health care, such as cancer treatment and management. These results should be interpreted in the context of individual risk factors and prioritize vaccination as a preventive measure to protect vulnerable populations. Public health strategies should continue to promote vaccination against COVID-19 along with maintaining access to essential non-COVID-19 health services. Our results may aid in chemotherapy decision making in the event of an outbreak of vaccine-resistant variant COVID-19.
