# Supplementary material for: Comparison of mortality in patients on chemotherapy or immunotherapy during and before COVID-19 pandemic. Multicenter matched cohort study in Argentina
Source: Rev Peru Med Exp Salud Publica. 2023 Jun 30;40(2):161–9. doi: 10.17843/rpmesp.2023.402.12519 (PMC10953664; doi:10.17843/rpmesp.2023.402.12519)
Supplement: Supplementary material. — Available in the electronic version of the RPMESP. [file rpmesp-40-02-12519-s001.docx]

**Material Suplementario**

COMPARACIÓN DE LA MORTALIDAD EN PACIENTES EN QUIMIOTERAPIA O INMUNOTERAPIA DURANTE Y ANTES DE LA PANDEMIA DE COVID-19. ESTUDIO MULTICÉNTRICO DE COHORTES EMPAREJADAS

**
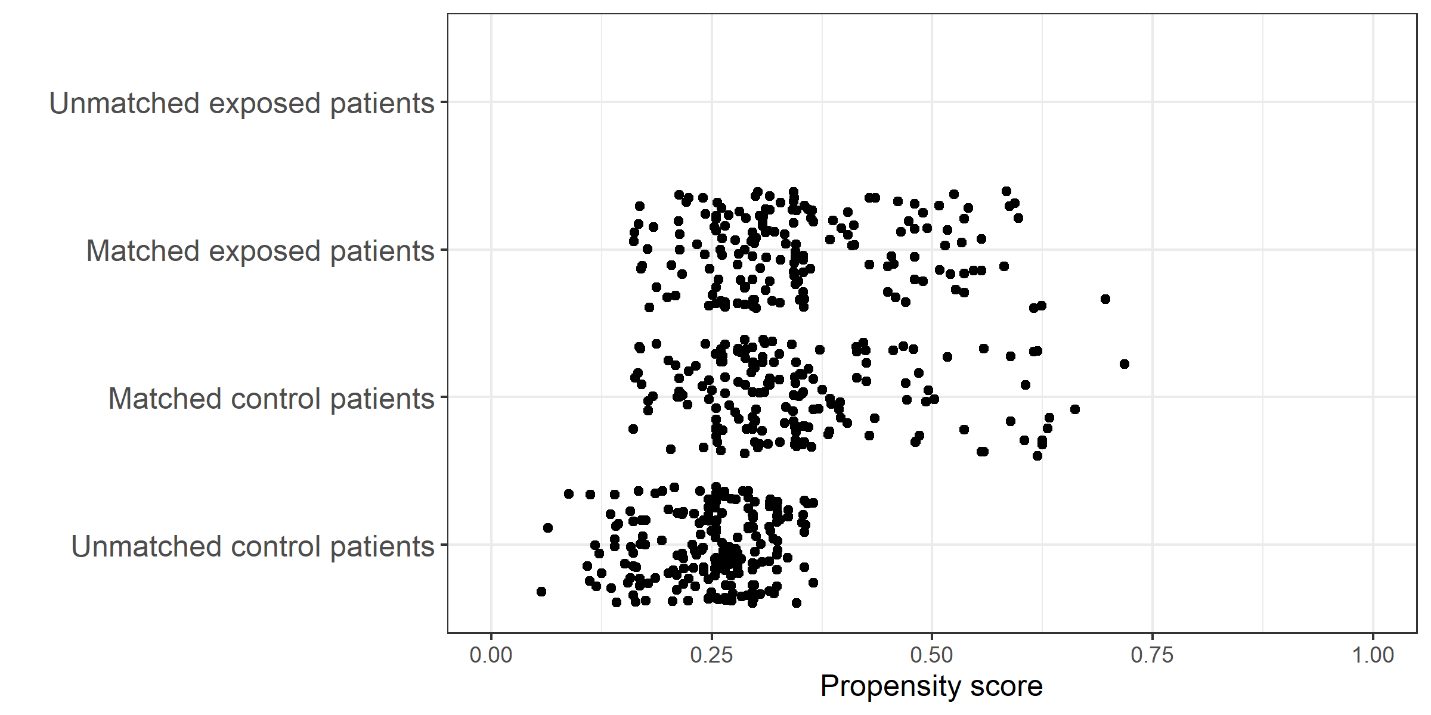
**

**Figure 1.** Distribution of propensity scores according to the exposure to pandemic.


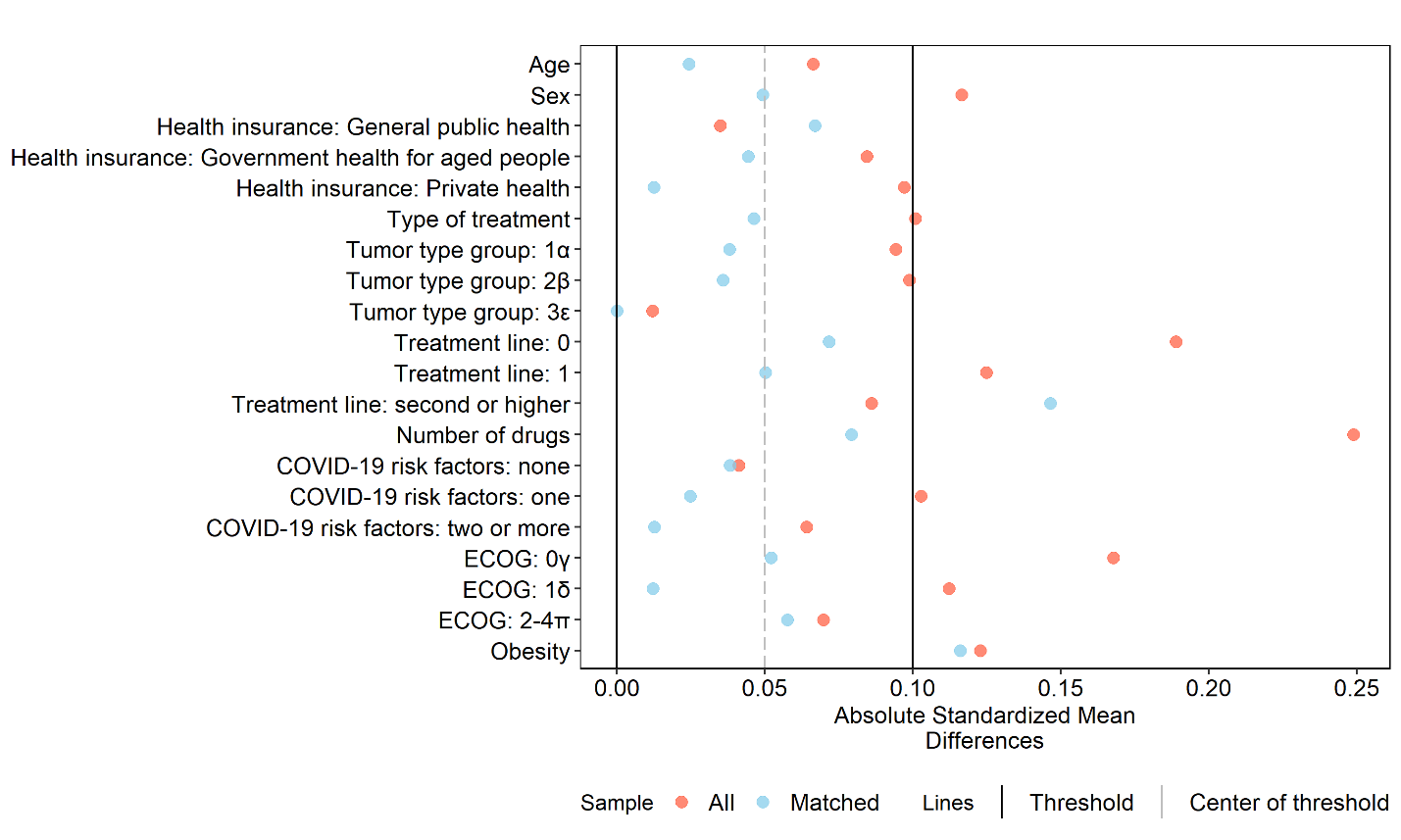
 **Figure 2.** Absolute Standard Mean Difference for each variable.

**Table 2.** Comparison of cohorts before matching.

| **Variables** | **Pandemic (N=169)** | | **Pre-Pandemic (N=377)** | | **P-Value** |
| --- | --- | --- | --- | --- | --- |
|  | n/N | % | n/N | % |  |
| Age | | | | |  |
| <=65 | 102/169 | 60.4% | 238/377 | 63.1% | 0.601 |
| >65 | 67/169 | 39.6% | 139/377 | 36.9% |  |
| Sex | | | | |  |
| Male | 63/169 | 37.3% | 118/377 | 31.3% | 0.203 |
| Female | 106/169 | 62.7% | 259/377 | 68.7% |  |
| Health insurance type | | | | |  |
| General Public Health | 23/167 | 13.8% | 51/376 | 13.6% | 0.580 |
| Government Health for Aged | 34/167 | 20.4% | 63/376 | 16.8% |  |
| Private health | 110/167 | 65.9% | 262/376 | 69.7% |  |
| Treatment Type | | | | |  |
| Chemotherapy | 154/166 | 92.8% | 360/377 | 95.5% | 0.275 |
| Immune therapy | 12/166 | 7.2% | 17/377 | 4.5% |  |
| Tumor type group | | | | |  |
| 1 ^a^ | 55/169 | 32.5% | 139/377 | 36.9% | 0.539 |
| 2,4,5 ^b^ | 77/169 | 45.6% | 154/377 | 40.8% |  |
| 3,6 ^c^ | 37/169 | 21.9% | 84/377 | 22.3% |  |
| Treatment line | | | | |  |
| (Neo)adjuvant | 76/169 | 45.0% | 135/376 | 35.9% | 0.129 |
| First line | 57/169 | 33.7% | 151/376 | 40.2% |  |
| Second or higher | 36/169 | 21.3% | 90/376 | 23.9% |  |
| Number of drugs in the treatment scheme | | | | | |
| One or two | 159/168 | 94.6% | 336/377 | 89.1% | 0.057 |
| Three or more | 9/168 | 5.4% | 41/377 | 10.9% |  |
| COVID-19 risk factors |  |  |  |  |  |
| None | 55/169 | 32.5% | 114/377 | 30.2% | 0.565 |
| 1^d^ | 60/169 | 35.5% | 152/377 | 40.3% |  |
| 2-6^d^ | 54/169 | 32% | 111/377 | 29.4% |  |
| ECOG | | | | |  |
| ECOG 0 | 50/168 | 29.8% | 141/377 | 37.4% | 0.205 |
| ECOG 1 | 100/168 | 59.5% | 204/377 | 54.1% |  |
| ECOG 2-4 | 18/168 | 10.7% | 32/377 | 8.5% |  |
| Obesity | | | | |  |
| No | 127/161 | 78.9% | 250/342 | 73.1% | 0.198 |
| Yes | 34/161 | 21.1% | 92/342 | 26.9% |  |

ECOG: Eastern Cooperative Oncology Group.

^a^ Testicular, Prostate, Breast, Gastrointestinal Stromal Tumor

^b^ Anal, Penile, Cervical, Colorectal, Soft tissue and bone sarcomas, Melanoma, Endometrial Renal, Head and Neck, Bladder, Ovarian, vulvovaginal, non-melanoma skin cancer

^c^ Biliopancreatic, Hepatocarcinoma, Lung, Mesothelioma, Esophageal, Gastric, Central Nervous System cancer

^d^ Number of COVID-19 risk factors: smoking or former smoking, respiratory diseases, arterial hypertension, heart diseases, diabetes, immunodeficiencies/chronic use of corticosteroids, chronic kidney disease, chronic liver disease.
