# Supplementary material for: Comparison of mortality in patients on chemotherapy or immunotherapy during and before COVID-19 pandemic. Multicenter matched cohort study in Argentina
Source: Rev Peru Med Exp Salud Publica. 2023 Jun 30;40(2):161–9. doi: 10.17843/rpmesp.2023.402.12519 (PMC10953664; doi:10.17843/rpmesp.2023.402.12519)
Supplement: Translation

# Translation: Comparación de la mortalidad en pacientes en quimioterapia o inmunoterapia durante y antes de la pandemia de COVID-19. Estudio multicéntrico de cohortes emparejadas en Argentina

**Authors:** Boris Itkin, Samanta Straminsky, Hernán Cáceres, Mariana Onassis, Agustín Emilio García, Laura Avanzi, Lucia Kaminszczik, Richard Serna Sejas, Mara Rapaccioli, Gustavo Billordo, Damián Lavaccara, Julián Lanzavecchia, Luz Gibbons, Eugenia Settecase, Ariel Bardach

**Affiliations:** 1 Hospital Juan A. Fernández, Buenos Aires, Argentina.; 2 Sanatorio Luis Pasteur, Catamarca, Argentina.; 3 Sanatorio Dr. Julio Méndez, Buenos Aires, Argentina.; 4 Policlínico Modelo de Cipolletti, Rio Negro, Argentina.; 5 Centro de Investigaciones en Epidemiología y Salud Pública (CIESP) Instituto de Efectividad Clínica y Sanitaria (IECS-CONICET), Buenos Aires, Argentina.

**Keywords:** Cáncer, Quimioterapia, Inmunoterapia, COVID-19, Mortalidad, Estudios de Cohortes

## Abstract

**Objetivos.:**

Comparar la mortalidad por todas las causas de pacientes oncológicos no vacunados que recibieron quimioterapia o inmunoterapia durante la pandemia, con aquellos tratados antes de la pandemia.

**Materiales y métodos.:**

Realizamos un estudio de cohortes en cuatro hospitales terciarios en Argentina. Pacientes ambulatorios con una neoplasia sólida de cualquier estadio en tratamiento citotóxico o inmune intravenoso fueron elegibles. La cohorte pandémica se enroló durante la fase inicial del brote y se comparó con una cohorte de un período anterior a la pandemia utilizando emparejamiento por puntuación de propensión (PSM, por sus siglas en inglés). Los sujetos se emparejaron por edad, sexo, seguro de salud, factores de riesgo para complicaciones graves por COVID-19, estado funcional, tipo de cáncer y tratamiento, línea de tratamiento e índice de masa corporal. La mortalidad por todas las causas se estimó en ambas cohortes después de seis meses de seguimiento.

**Resultados.:**

169 pacientes fueron reclutados entre abril y agosto de 2020 para la cohorte pandémica y 377 para la cohorte prepandémica en el mismo período de 2019, 168 pacientes fueron emparejados. Luego de la PSM, la mortalidad por todas las causas fue del 17,9% en la cohorte pandémica y del 18,5% en la cohorte prepandémica, Riesgo Relativo: 0,97 (intervalo de confianza al 95 %: 0,61-1,52; p=0,888). En la cohorte pandémica, 30/168 pacientes fallecieron, ninguno por infección por COVID-19.

**Conclusiones.:**

No hemos observado un aumento de mortalidad en pacientes ambulatorios no vacunados en tratamiento oncológico endovenoso activo durante la pandemia por COVID-19.

## INTRODUCCIÓN

El cáncer es la segunda causa de mortalidad en América Latina y el Caribe (ALC), con más de 700,000 muertes en 2020 ^1^. Las importantes dificultades que esta carga ya suponía para la región se agravaron aún más con la contingencia de la pandemia por COVID-19 ^2^. Todo el espectro de la atención oncológica, desde la prevención hasta la supervivencia, se ha visto afectado por el predominio de la pandemia por COVID-19, lo que plantea a los proveedores, un nuevo reto a la hora de sopesar los beneficios de los tratamientos contra el cáncer y el riesgo de su administración en medio de la pandemia. Un primer estudio demostró que la quimioterapia intravenosa (IV) se asociaba a un mayor riesgo de complicaciones graves en pacientes con cáncer infectados por el virus SARS-CoV-2 ^3^. Se ha sugerido que, la inmunosupresión inducida por la quimioterapia y la enfermedad, la toxicidad pulmonar relacionada con el tratamiento, la necesidad de hospitalización con el riesgo asociado de infecciones nosocomiales junto con la saturación del sistema sanitario, el distanciamiento social y las cuestiones económicas son factores que pueden influir en el riesgo de complicaciones graves y muerte de los pacientes con cáncer en tratamiento anticanceroso activo ^4^^-^^6^. Esto se ha reflejado en las recomendaciones de sociedades médicas y expertos que sugieren la sustitución de la quimioterapia por fármacos endocrinos, el uso de protocolos de tratamiento con un menor número de ciclos y la sustitución de fármacos intravenosos (IV) por análogos orales, lo que conlleva modificaciones de la práctica de tratamiento con un impacto potencial en los resultados oncológicos ^4^^,^^5^^,^^7^^,^^8^. Durante la pandemia, también hubo preocupación por la seguridad de la inmunoterapia debido a una incidencia significativa de neumonitis inmunorrelacionada que requirió corticosteroides e inmunosupresores ^6^^,^^9^.

Muchos estudios en el campo de la COVID-19 y el cáncer se han centrado en los resultados de pacientes oncológicos con COVID-19 demostrada en comparación con aquellos sin cáncer o COVID-19. Por lo general, tenían como fuente de datos a sujetos positivos a la COVID-19 ^10^^,^^11^. Sin embargo, este enfoque puede ser menos apropiado para evaluar el riesgo incremental de muerte asociado a la pandemia por COVID-19 en la población general de pacientes oncológicos ambulatorios que reciben un tratamiento IV activo. Por otro lado, la estimación de la mortalidad específica por COVID-19 como indicador del impacto de la pandemia en la salud de los pacientes oncológicos puede ser difícil o poco fiable debido, por ejemplo, a la clasificación errónea de la causa de muerte, las variaciones en la realización y notificación de las pruebas de COVID-19 y el fenómeno de desplazamiento de la enfermedad ^12^. La mortalidad por todas las causas es una medida que engloba todas las muertes por cualquier causa y permite superar los problemas citados proporcionando una métrica del impacto de la pandemia en la mortalidad general ^12^.

La mayor parte de la investigación sobre COVID-19 y el cáncer procede de países desarrollados ^12^. Sin embargo, no está claro hasta qué punto los resultados de estos estudios son generalizables a las naciones en desarrollo. En ALC, el panorama sanitario previo a la pandemia ya estaba plagado de sistemas sanitarios fragmentados y con financiación insuficiente, desigualdades en el acceso a una atención de calidad y una disponibilidad reducida de pruebas y vacunas contra la COVID-19. En toda la región, se aplicaron ampliamente los cierres y las cuarentenas ^13^. Nuestro objetivo fue comparar la mortalidad de pacientes oncológicos no vacunados que recibieron quimioterapia o inmunoterapia durante la pandemia, con aquellos tratados antes de la pandemia.

**MENSAJES CLAVE**

Motivación para realizar el estudio. El impacto de la pandemia por COVID-19 en el riesgo de muerte en pacientes con cáncer en quimioterapia e inmunoterapia es controversial. Los estudios publicados comparaban, principalmente, a pacientes en tratamiento anticanceroso con los que no lo recibían o pacientes con cáncer y COVID-19 positivo con COVID-19 negativo. Pocos estudios se realizaron en países en vías de desarrollo.

Principales hallazgos. No se ha observado un aumento de la mortalidad en pacientes ambulatorios no vacunados en tratamiento oncológico endovenoso activo durante la pandemia por COVID-19.

Implicancias. Este es el primer estudio de cohortes emparejado por puntuación de propensión que evalúa el impacto de la pandemia por COVID-19 en la población de pacientes oncológicos no vacunados que reciben tratamiento anticanceroso intravenoso.

## MATERIALES Y MÉTODOS

### Diseño y entorno del estudio

Realizamos un estudio de cohortes prospectivo multicéntrico que tuvo como referencia a una cohorte retrospectiva emparejada por puntuación de propensión del año anterior al inicio de la pandemia. Formamos un grupo colaborativo en el que participaron cuatro hospitales terciarios de Argentina, uno público y tres privados ubicados en diferentes regiones geográficas. En la cohorte pandémica, reclutamos prospectivamente a pacientes en las Unidades de Día de los centros participantes entre el 15 de abril de 2020 y el 26 de agosto de 2020. La cohorte de referencia (cohorte prepandémica) se construyó utilizando los datos de las historias clínicas de los pacientes tratados en los mismos centros en el periodo emparejado de 2019 antes del inicio de la pandemia.

### Participantes

Para ensamblar la cohorte pandémica, utilizamos un muestreo probabilístico simple dentro de cada centro. Los sujetos elegibles para participar fueron asignados aleatoriamente a participar o no utilizando un generador de números aleatorios en una oficina virtual de aleatorización remota. Para la cohorte histórica, se utilizó un muestreo por conveniencia. Los pacientes de cualquier edad y sexo con un diagnóstico histológicamente probado de una neoplasia sólida que recibieron terapia IV citotóxica o inhibidores de puntos de control inmunitario (ICI, por sus siglas en inglés) como agente único o en cualquier combinación fueron elegibles independientemente de la disponibilidad de la prueba de COVID-19 y sus resultados. Además, se incluyeron los pacientes en tratamiento activo de quimiorradiación concurrente y los que recibían una combinación de quimioterapia IV y fármacos orales, sean diana o citotóxicos. Se excluyeron los pacientes que recibieron terapia hormonal o diana sin fármacos citotóxicos o ICI concomitantes. El periodo de seguimiento fue de seis meses.

### Tamaño de muestra

En el momento del diseño del estudio, en marzo de 2020, se informó de un aumento significativo de acontecimientos clínicos graves en pacientes con cáncer y COVID-19 positivo que recibieron quimioterapia en comparación con los que no la recibieron, con un Odds ratio de 5,34 (intervalo de confianza al 95% [IC 95%]: 1,8 - 16,2) ^3^. Debido a que no se disponía de ningún otro estudio relevante sobre el tema, tomamos como referencia el límite inferior de este IC. Con 168 pacientes por cohorte, el estudio tenía una potencia estadística del 87% para detectar un aumento del 70% de la mortalidad en la cohorte pandémica en comparación con el periodo sin pandemia.

### Fuente de datos y mediciones

Se recopilaron datos sobre las características clínicas, demográficas, relacionadas con el tratamiento y los resultados de supervivencia a partir de historias clínicas (convencionales y electrónicas). Se contactó por teléfono a los pacientes y sus familiares cuando fue necesario. Los tumores se estadificaron según el *American Joint Committee on Cancer Staging Manual*, 8.ª edición ^14^. Debido a una importante escasez de hisopos y pruebas serológicas de COVID-19 en Argentina al momento del diseño del estudio, en marzo de 2020, y hasta el final del período de seguimiento, estas pruebas no fueron obligatorias, sino que se realizaron según las indicaciones clínicas y la disponibilidad. Ningún paciente recibió la vacuna contra la COVID-19, ya que, el período de seguimiento finalizó antes de que estuviera disponible en Argentina.

### Variables

*Exposición y desenlaces*

El estado de COVID-19 no puede conocerse en un solo individuo en un momento dado, y el pronóstico de vida no es sólo una función de la gravedad clínica de la COVID-19, sino que, potencialmente, depende de una compleja interacción de muchos factores muy variables en el tiempo y difíciles de estimar. Entre ellos, se incluyen la posibilidad de contagio de COVID-19, la recuperación de las complicaciones del tratamiento contra el cáncer y la COVID-19, los retrasos o cancelaciones de los tratamientos oncológicos, la disponibilidad de recursos del sistema sanitario, el apoyo familiar y social, la adherencia individual a las medidas de restricción y la capacidad para afrontar algunos costes extraordinarios relacionados con la salud, entre otros. Por lo tanto, definimos la «inmersión» de los pacientes en el entorno pandémico como exposición.

El resultado primario de nuestro estudio fue la mortalidad por cualquier causa al final del periodo de seguimiento en ambas cohortes, definida como el número de muertes sobre el número total de participantes. La tasa de complicaciones graves fue el resultado secundario. No se calculó la supervivencia en función del tiempo porque no se disponía de datos individuales en la cohorte prepandémica.

*Emparejamiento de cohortes y covariables*

Debido a las posibles diferencias entre cohortes en las covariables de posible importancia pronóstica, utilizamos el emparejamiento por puntuación de propensión (PSM, por sus siglas en inglés) para controlar los factores de confusión conocidos. Para la especificación del modelo, realizamos previamente un análisis de regresión de Cox utilizando los datos de supervivencia de la cohorte pandémica tras cuatro meses de seguimiento. Encontramos variables significativamente asociadas con la probabilidad de supervivencia: *Eastern Oncology Group* (ECOG) *Performance Status*, línea de tratamiento e índice de masa corporal (IMC) (datos propios no publicados disponibles bajo petición). Además, incluimos como covariable al número de factores de riesgo de complicaciones graves y muerte en pacientes con infección por el virus SARS-CoV-2 ^5^. Asimismo, incluimos covariables que influyen potencialmente en la probabilidad de muerte, como la edad, el sexo, el tipo de seguro médico, el número de fármacos del régimen de tratamiento y el tipo de tumor. Para tratar las diferencias en el pronóstico según el subtipo de cáncer, clasificamos los tumores en tres grupos pronósticos según la mediana de supervivencia a cinco años esperada en todos los estadios (Tabla 1) ^15^^,^^16^.

**Tabla 1:** Grupos pronósticos por subtipo de cancer.

Grupo pronóstico	Subtipo de cáncer
1	Testicular, próstata, mama, GIST
2	Anales, peneanos, cervicales, colorrectales, sarcomas de partes blandas y óseos
3	Biliopancreático, Hepatocarcinoma, Pulmón, Mesotelioma, Esófago
4	Melanoma, Endometrio Renal, Cabeza y Cuello, Vejiga
5	Ovario, vulvovaginal, piel no melanoma
6	Gástrico, SNC

GIST: *gastrointestinal stromal tumor*; SNC: sistema nervioso central.

En cuanto al estadio tumoral, asumimos que la probabilidad de muerte a corto plazo podía tenerse en cuenta dividiendo a los pacientes en categorías de enfermedad no metastásica y avanzada. En este caso, la variable «línea de tratamiento» incluye la información pronóstica relevante relacionada con el estadio tumoral, ya que los tratamientos neo(adyuvantes) coinciden con los estadios no metastásicos. La lista de covariables incluidas en la puntuación de propensión se muestra en el Material Suplementario.

### Análisis estadístico

Hubo un porcentaje moderado (7,5%) de valores omitidos en la variable IMC, mientras que el resto de covariables mostraron una omisión baja (0,0%-0,5%). Se imputaron los datos que faltaban con el método de imputaciones múltiples por ecuaciones encadenadas (MICE, por sus siglas en inglés) debido a su aceptación universal, flexibilidad y buen rendimiento tanto en variables continuas como categóricas ^17^. Primero, se identificaron las variables con valores perdidos y se crearon cadenas de ecuaciones para cada una de estas variables. Estas cadenas consisten en modelos de regresión que predicen un valor omitido en una variable basándose en los valores observados en otras variables. Se actualizaron iterativamente las imputaciones de cada variable mediante un muestreo secuencial de las distribuciones condicionales de las demás variables de la cadena. Este proceso se repitió varias veces para garantizar la convergencia y la estabilidad de las imputaciones.

Las puntuaciones de propensión se estimaron mediante regresión logística ^18^. Se utilizó el emparejamiento óptimo uno a uno con un método de sustitución basado en el algoritmo de optimización de flujo de red *Ford-Fulkerson*, ya que se espera que supere al emparejamiento uno a uno codicioso ^19^^-^^21^. Se utilizaron la diferencia de medias estandarizada (DME) y los gráficos de densidad para evaluar el equilibrio de los grupos. Como la DME entre los grupos emparejados estaba por debajo del umbral de 0,1, concluimos que el equilibrio era satisfactorio. Las cohortes emparejadas se compararon con una prueba chi-cuadrado. Se utilizó el paquete Matchlt para la aplicación del método ^22^.

Se comunicó la tasa de mortalidad de ambas cohortes y el riesgo relativo (RR) crudo y ajustado con un IC al 95%. Estimamos el RR ajustado mediante regresión logística con función de enlace logarítmico de distribución de Poisson. Se ajustó por las variables incluidas en el PSM. Se utilizó el método binomial para estimar la tasa de complicaciones graves con un IC al 95%. Todas las pruebas estadísticas se realizaron con un alfa de 0,05 utilizando el software de código abierto R 4.0.3.

### Aspectos éticos

Los Comités de Ética de todos los centros participantes aprobaron el estudio (fecha: 08/04/2020, código de registro: 1234, número: DI-2020-353-CABA-HGAJF), y todos los pacientes de la cohorte pandémica firmaron un consentimiento informado.

## RESULTADOS

### Cohortes y emparejamiento

Antes de la PSM, el número de pacientes en las cohortes pandémica y prepandémica era de 169 y 377, respectivamente (Figura 1). Al inicio del estudio, la edad media era de 59,2 años (desviación estándar [DE]: 13,7 años) en la cohorte pandémica y de 59,4 años (DE: 13,5 años) en la prepandémica. Las mujeres representaban el 62,7% y el 68,7%, respectivamente. La quimioterapia fue el tratamiento más común, 92,6% en la pandemia y 95,5% en la cohorte prepandémica, y una pequeña proporción de pacientes recibieron ICI (7,2% y 4,5%, respectivamente). Las terapias (neo)adyuvantes y de primera línea fueron las más frecuentes, 78,7% y 77,3%, respectivamente. La proporción de pacientes con dos o más factores de riesgo de complicaciones graves de infección por el virus SARS-CoV-2 fue del 32,1% y 32,7%, respectivamente. En el Material Suplementario se resumen las características clínicas, demográficas y relacionadas con el tratamiento de los participantes. La mediana de seguimiento fue de 6,0 meses en ambas cohortes. En la cohorte pandémica, el hisopo nasal del 4,8% dio positivo para COVID-19. En la cohorte pandémica, se perdió el seguimiento de uno de los 169 pacientes y no se dispuso de datos sobre el resultado principal. En la cohorte prepandémica, no se perdió el seguimiento de ninguno de los 377 pacientes.

**Figura 1:** Flujo de pacientes incluidos en el estudio.

Como resultado de la PSM, 168 sujetos fueron emparejados. En la cohorte prepandémica, 209 sujetos quedaron fuera, mientras que sólo un sujeto quedó fuera de la cohorte pandémica. Las características de los pacientes en las cohortes pandémica y prepandémica tras el emparejamiento se muestran en la Tabla 2. No se encontraron diferencias estadísticamente significativas entre las cohortes según las covariables incluidas en el modelo. En el Material Suplementario se muestra la distribución de las puntuaciones de propensión tanto en las cohortes emparejadas como en los sujetos no emparejados. La DME absoluta para cada covariable se muestra en el Material Suplementario.

**Tabla 2:** Comparación de cohortes tras el emparejamiento.

Variables	Cohorte				Valor de p
	Pandémica (N=168)		Prepandémica (N=168)		
	n	%	n	%	
Edad					
65 o menor	101	60,1	100	59,5	1,000
Mayor de 65 años	67	39,9	68	40,5	
Sexo					
Masculino	62	36,9	66	39,3	0,736
Femenino	106	63,1	102	60,7	
Tipo de Seguro de salud					
Salud pública general	25	14,9	22	13,1	0, 811
Sanidad para adultos mayores	34	20,2	38	22,6	
Salud privada	109	64,9	108	64,3	
Tipo de tratamiento					
Quimioterapia	156	92,9	154	91,7	0,838
Inmunoterapia	12	7,1	14	8,3	
Grupo de tipo de tumor					
1 ^a^	55	32,7	48	28,6	0,709
2,4,5 ^b^	77	45,8	82	48,8	
3,6 ^c^	36	21,4	38	22,6	
Linea de tratamiento					
(Neo)adyuvante	76	45,2	74	44,0	0,924
Primera linea	57	33,9	56	33,3	
Segunda o tercera	35	20,8	38	22,6	
Número de medicamentos					
Uno o dos	159	94,6	157	93,5	0,818
Tres o más	9	5,4	11	6,5	
Factores de riesgo para COVID-19					
Ninguno	54	32,1	47	28,0	0,677
1 ^d^	60	35,7	66	39,3	
2-6 ^d^	54	32,1	55	32,7	
ECOG					
0	50	29,8	50	29,8	0,939
1	100	59,5	98	58,3	
2-4	18	10,7	20	11,9	
Obesidad					
No	132	78,6	134	79,8	0,893
Sí	36	21,4	34	20,2	

ECOG: *Eastern Cooperative Oncology Group.*

a Tumor testicular, de próstata, de mama, del estroma gastrointestinal.

b Anal, peneano, cervical, colorrectal, sarcomas de tejidos blandos y óseos, melanoma, renal, cabeza y cuello, vejiga, ovario, vulvovaginal, cáncer de piel no melanoma.

c Cáncer biliopancreático, hepatocarcinoma, pulmón, mesotelioma, esofágico, gástrico, sistema nervioso central.

d Número de factores de riesgo COVID-19: tabaquismo o ex tabaquismo, enfermedades respiratorias, hipertensión arterial, cardiopatías, diabetes, inmunodeficiencias/uso crónico de corticosteroides, enfermedad renal crónica, enfermedad hepática crónica.

### Mortalidad y admisiones

Tras la PSM, la tasa de mortalidad por todas las causas fue de 30/168 (17,9%, IC 95%: 12,4% - 24,5%) en la cohorte pandémica y 31/168 (18,5%, IC 95%: 12,9% - 25,2%) en la cohorte prepandémica (RR: 0,97, IC 95%: 0,61 - 1,52; p=0,888 y RR ajustado [RRa]: 1,07, IC 95%: 0,63 - 1,79; p=0,810). La tasa de complicaciones graves en la cohorte pandémica fue de 8/167 (4,8%, IC 95%: 2,1% - 9,2%). De los 30 pacientes fallecidos en la cohorte pandémica, 29 murieron por progresión del cáncer, 0 pacientes (0,0%) por infección por el virus SARS-CoV-2, y un paciente (3,3%, IC 95%: 0,0% - 1,7%) por otra causa. No se observó ninguna asociación estadísticamente significativa entre el estado vital al final del periodo de seguimiento y la positividad a COVID-19 (valor de p de la prueba exacta de Fisher igual a 1). En la cohorte pandémica 8/167 (4,8%, IC 95%: 2,1% - 9,2%) de los pacientes tuvieron un ingreso en una Unidad de Cuidados Intensivos (UCI), 7/167 (4,2%, IC 95%: 1,7% - 8,4%) en una Unidad de Alta Dependencia, y 47/167 (28,1%, IC 95%: 21,5% - 35,6%) en una sala ordinaria. No se encontró ninguna relación estadísticamente significativa entre los ingresos y la positividad de la prueba COVID-19 (valor de p de la prueba exacta de Fisher igual a 1).

### Análisis de sensibilidad

Para evaluar si nuestros resultados se ven influidos por la inclusión de pacientes en tratamiento inmunológico, realizamos un análisis de sensibilidad eliminándolos de las cohortes. Tras el PSM, la mortalidad por todas las causas fue de 28/56 (17,9%) y 27/154 (17,5%) en las cohortes pandémica y prepandémica, respectivamente; RRa: 1,07 (IC 95%: 0,57- 1,99; p=0,329).

### Valores perdidos

La variable IMC presentaba una proporción moderada (7,5%) de valores perdidos, mientras que el resto de las covariables mostraban un nivel bajo de valores perdidos (entre el 0,0% y el 0,5%).

## DISCUSIÓN

A diferencia de la mayoría de los estudios publicados que suelen centrarse en los resultados de los pacientes con cáncer y COVID-19 positivo, el presente estudio abordó el riesgo de la administración de terapia oncológica IV en el entorno pandémico de forma diferente. Comparamos la mortalidad de pacientes oncológicos en quimioterapia IV activa o inmunoterapia independientemente de su estado de COVID-19 con una cohorte emparejada del periodo pandémico y no pandémico. El estudio se realizó en un país en vías de desarrollo antes del uso de vacunas ^23^.

En nuestro análisis, tras equilibrar los posibles factores de confusión (edad, sexo, seguro de salud, tipo de fármaco, grupo de tumor primario, línea de tratamiento, número de fármacos, factores de riesgo, estado funcional ECOG y obesidad), no se encontraron diferencias estadísticamente significativas en la tasa de mortalidad por todas las causas de los pacientes tratados durante la pandemia (17,9%) frente a los tratados antes de la pandemia (18,5%) (RR: 0,97; IC 95%: 0,61-1,52, p=0,888).

Una tasa de mortalidad de casi el 18% en un periodo de seguimiento de seis meses en nuestro estudio parece superior en comparación con la tasa de mortalidad del 4% al 8% notificada en un gran estudio de cohortes del Reino Unido. La inclusión en el estudio británico de pacientes en tratamiento con fármacos diana y quimioterapia oral con un pronóstico prioritariamente mejor podría servir de posible explicación. Sin embargo, no pueden descartarse diferencias en la calidad de la asistencia ^24^.

Hasta la fecha, muchos estudios caracterizados por importantes disimilitudes en su diseño, tamaño, población, tiempo de seguimiento, definiciones, métodos de diagnóstico y eventos de interés evaluaron la mortalidad en pacientes oncológicos durante la pandemia. Muchos eran retrospectivos y comparaban pacientes oncológicos y COVID-19 positivos en tratamiento anticanceroso con aquellos sin ningún tratamiento activo. Varias revisiones sistemáticas intentaron resumir las pruebas disponibles. Los meta-análisis realizados por Wang *et al.*, Yekeduz *et al.*, Park *et al.* y Wu *et al.* incluyeron en su mayoría estudios retrospectivos de China, Europa y Norteamérica, con un solapamiento parcial ^25^^-^^27^. Compararon la mortalidad en pacientes COVID-19 positivo con neoplasias malignas sólidas y hematológicas en quimioterapia en relación con el brazo control, que normalmente combina pacientes sin tratamiento anticanceroso con tratamientos no citotóxicos. Todos ellos hallaron incrementos de la mortalidad en el brazo de quimioterapia. Por el contrario, Lin *et al.* concluyeron que la quimioterapia y la ICI no aumentaban la mortalidad en pacientes con cáncer y COVID-19 ^28^^,^^29^.

Un amplio estudio del Reino Unido realizado por Russel *et al.*, similar a nuestro trabajo, utilizó como grupo de control a una cohorte de un periodo prepandémico (2019) ^30^. Además de pacientes con fármacos citotóxicos e inmunoterapia, el estudio incluyó pacientes con terapia biológica y terapia dirigida. Las cohortes no estaban equilibradas por PSM. Los autores descubrieron que la quimioterapia sistémica durante la primera oleada de la pandemia por COVID-19 no aumentó la mortalidad en pacientes con tumores sólidos en relación con el periodo prepandémico. Se observaron tasas bajas de infección y mortalidad por COVID-19 en consonancia con nuestros hallazgos ^30^.

Para conciliar las conclusiones controvertidas de los análisis publicados, planteamos la hipótesis de que, aunque los fármacos citotóxicos todavía pueden aumentar la mortalidad en la población general de pacientes con cáncer, podría ser difícil de detectar en entornos con bajas tasas de prevalencia y transmisión. El tipo de neoplasia, hematológica o sólida, también puede ser importante. Sería necesario un estudio de gran tamaño para probar esta hipótesis. Las investigaciones futuras deberían considerar la realización de estudios con mayor granularidad de datos para evaluar mejor los subgrupos relevantes.

En nuestro estudio, la ausencia de exceso de mortalidad en la cohorte pandémica, discrepante con muchas publicaciones anteriores, admite varias explicaciones potenciales. Por ejemplo, el impacto global de la pandemia por COVID-19 en la población general de pacientes con cáncer puede ser menor que en aquellos con infección clínicamente manifiesta o pruebas de COVID-19 positivas. La mayor parte de los estudios publicados evaluaron estas últimas poblaciones. Si la infección por el virus SARS-CoV-2 en la población general de pacientes con tumores sólidos es predominantemente asintomática, la mortalidad global no aumentará a menos que la infección sea altamente letal. En este sentido, una baja carga de pandemias en Argentina durante el período de datos del estudio podría ser, en apariencia, una explicación tentadora. Sin embargo, en Argentina se registraron más de 2 millones de casos de infección por el virus SARS-CoV-2 en el período mencionado ^31^. El verdadero número acumulado de casos puede ser aún mayor, si se tiene en cuenta que la capacidad de análisis era limitada incluso en los países de altos ingresos ^32^.

Los puntos fuertes de nuestro estudio son la inscripción prospectiva en la cohorte pandémica, la participación de múltiples centros distribuidos ampliamente por todo el territorio nacional, la inclusión de sistemas sanitarios públicos y privados, y el emparejamiento de cohortes por puntuación de propensión. Sin embargo, nuestro estudio tiene varias limitaciones. La comparación de una cohorte reclutada prospectivamente con otra retrospectiva es la principal fuente de sesgo y la principal limitación de nuestro estudio. La utilización de PSM es una forma de reducirlo. Otra limitación importante es el pequeño tamaño de la muestra. El estudio se potenció para detectar sólo grandes incrementos en la mortalidad, ya que las suposiciones realizadas durante el proceso de diseño del estudio al principio de la pandemia se vieron influidas por la información de un impacto potencialmente muy significativo de la COVID-19 en la salud de los pacientes oncológicos ^3^^,^^5^. La proporción de sujetos sometidos a la prueba de la COVID-19 fue baja. Probablemente se deba a una disponibilidad de pruebas significativamente reducida en Argentina durante la primera fase de la pandemia junto con una baja tasa de infección sintomática por el virus SARS-CoV-2 en nuestro estudio. Nuestra hipótesis es que esto último podría ser el resultado de una menor diseminación viral en la población de estudio debido a los encierros masivos y estrictos, aunque su adherencia disminuyó gradualmente con el tiempo debido a un fenómeno de fatiga ^13^. El corto período de seguimiento es otra limitación de nuestro trabajo. El seguimiento se interrumpió una vez iniciada la vacunación en Argentina. Es posible que nuestros hallazgos no sean representativos de toda la población de pacientes oncológicos ni aplicables a diferentes regiones geográficas o períodos del calendario.

De acuerdo con una revisión realizada por los autores del presente trabajo, este es el primer estudio de cohortes emparejado por puntuación de propensión que evalúa el impacto de la pandemia por COVID-19 en la población de pacientes oncológicos no vacunados que reciben tratamiento anticanceroso IV en comparación con un periodo sin pandemia. A pesar de las limitaciones comentadas, nuestro estudio proporciona cierto apoyo empírico a la idea de que la quimioterapia IV y la inmunoterapia podrían administrarse de forma segura durante la pandemia antes del uso de la vacuna contra la COVID-19 en un país de ingresos medios y contribuye al debate sobre la interacción entre la pandemia por COVID-19 y la atención oncológica. Nuestros resultados sugieren que, incluso en medio de una pandemia, sigue siendo crucial priorizar y garantizar un acceso adecuado a la atención sanitaria no relacionada con la COVID-19, como el tratamiento y la gestión del cáncer. Estos resultados deben interpretarse en el contexto de los factores de riesgo individuales y dar prioridad a la vacunación como medida preventiva para proteger a las poblaciones vulnerables. Las estrategias de salud pública deben seguir promoviendo la vacunación contra la COVID-19 junto con el mantenimiento del acceso a los servicios sanitarios esenciales no relacionados con la COVID-19. Nuestros resultados pueden ayudar en la toma de decisiones sobre la quimioterapia en el caso de un brote de la variante COVID-19 resistente a la vacuna.
